# Mosaic dysfunction of mitophagy in mitochondrial muscle disease

**DOI:** 10.1016/j.cmet.2021.12.017

**Published:** 2022-02-01

**Authors:** Takayuki Mito, Amy E. Vincent, Julie Faitg, Robert W. Taylor, Nahid A. Khan, Thomas G. McWilliams, Anu Suomalainen

**Affiliations:** 1 Research Program of Stem Cells and Metabolism, Faculty of Medicine, Biomedicum Helsinki, University of Helsinki, 00290 Helsinki, Finland; 2 Wellcome Centre for Mitochondrial Research, Translational and Clinical Research Institute, Faculty of Medical Sciences, Newcastle University, Newcastle upon Tyne NE2 4HH, UK; 3 NHS Highly Specialised Service for Rare Mitochondrial Disorders, Newcastle upon Tyne Hospitals NHS Foundation Trust, Newcastle upon Tyne NE2 4HH, UK; 4 Department of Anatomy, Faculty of Medicine, University of Helsinki, 00290 Helsinki, Finland; 5 Helsinki University Hospital, HUSlab, 00290 Helsinki, Finland

**Keywords:** mitophagy, mitochondrial myopathy, ragged-red fibers, centrally nucleated fibers, *mito*-QC, lysosome, patient, mitochondrial disease, SBFSEM

## Abstract

Mitophagy is a quality control mechanism that eliminates damaged mitochondria, yet its significance in mammalian pathophysiology and aging has remained unclear. Here, we report that mitophagy contributes to mitochondrial dysfunction in skeletal muscle of aged mice and human patients. The early disease stage is characterized by muscle fibers with central nuclei, with enhanced mitophagy around these nuclei. However, progressive mitochondrial dysfunction halts mitophagy and disrupts lysosomal homeostasis. Interestingly, activated or halted mitophagy occur in a mosaic manner even in adjacent muscle fibers, indicating cell-autonomous regulation. Rapamycin restores mitochondrial turnover, indicating mTOR-dependence of mitochondrial recycling in advanced disease stage. Our evidence suggests that (1) mitophagy is a hallmark of age-related mitochondrial pathology in mammalian muscle, (2) mosaic halting of mitophagy is a mechanism explaining mosaic respiratory chain deficiency and accumulation of pathogenic mtDNA variants in adult-onset mitochondrial diseases and normal aging, and (3) augmenting mitophagy is a promising therapeutic approach for muscle mitochondrial dysfunction.

## Highlights

• Muscle shows mosaic presentation of mitophagy, high in central nuclear fibers
• Increased mitophagy is an early response to muscle mitochondrial pathology
• Stalled mitophagy in advanced pathology explains accumulation of disease signs
• Rapamycin augments mitophagy, suggesting mitophagy activators as therapy tools

## Graphical abstract

**Figure:**
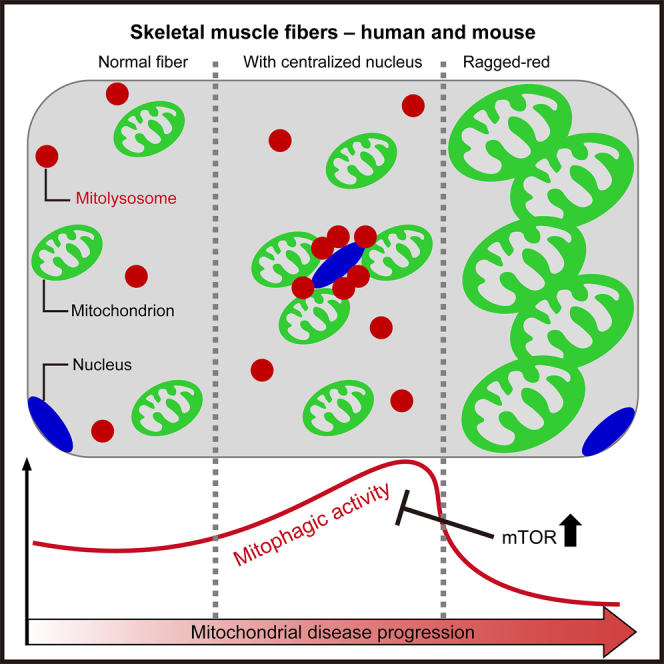

## Teaser

Mito et al. reveal a mosaic pattern of mitophagy activation in mitochondrial myopathy muscle fibers. Increased mitophagy zones surround central nuclei, while mitophagy halts in advanced disease. The findings provide evidence as to why mtDNA variants accumulate in muscle. Rescue of mitophagy by rapamycin suggests mitophagy activation as a therapeutic approach for mitochondrial myopathies.

## Introduction

Mitochondrial dysfunction is an integral component of degenerative diseases, from neurodegeneration to disorders of sensory or endocrine organs, heart and skeletal muscle, as well as normal aging (Suomalainen and Battersby, 2018). Indeed, up to 30% of muscle fibers in nonagenarians were found to be deficient for the mitochondrial respiratory chain (RC) activities (Bua et al., 2006) associated with the progressive accumulation of pathogenic mitochondrial DNA (mtDNA) variants. Such RC-deficient fibers, some with abundant ultrastructurally abnormal mitochondria (ragged-red fibers; RRFs), are also hallmarks of mitochondrial muscle disease and typically harbor high amounts of pathogenic mtDNA variants. The similarities suggest related mechanisms in primary and secondary mitochondrial dysfunction. Furthermore, muscle fibers with centralized nuclei (CNFs) are common in degenerative muscle diseases of different kinds (Olson et al., 1972), but the underlying molecular mechanisms have remained unclear. Why dysfunctional mitochondria are not destined to be recycled in the affected fibers, why pathogenic mtDNA variants accumulate in muscle cells, why nuclei centralize in muscle disease, and the mechanisms that contribute to progression of mitochondrial pathogenesis and muscle fatigue in aging remain unknown.

The removal of damaged organelles is essential to prevent cellular dysfunction and can occur by non-selective turnover (macroautophagy) or targeted elimination of organelles (selective autophagy) (Suomi and McWilliams, 2019). Mitophagy refers to the selective elimination of damaged mitochondria by autophagy, characterized originally in cultured cells under hypoxia or after pharmacological dissipation of mitochondrial membrane potential (Narendra et al., 2008; Twig et al., 2008; Zhang et al., 2008). The recent advent of optical reporter systems to monitor mitophagy *in vivo* has transformed our understanding of this process in mammalian physiology (Kuma et al., 2017; Montava-Garriga and Ganley, 2020; Palikaras et al., 2018; Suomi and McWilliams, 2019). One of the best characterized mitophagy reporter systems is *mito*-QC, a tandem mCherry-GFP protein localized to the outer mitochondrial membrane. During mitophagy, encapsulated mitochondria within autophagosomes fuse with acidic lysosomal compartments. The acid labile GFP fluorescence is quenched in the lysosome, and the remaining mCherry signal enables quantitative profiling of mitophagy *in vitro* and *in vivo* (Allen et al., 2013; McWilliams et al., 2016). Application of *mito*-QC variants on mice has successfully revealed the complex nature of mitophagy in tissues in a range of defined contexts. (Hoshino et al., 2019; McWilliams et al., 2016, 2018a, 2018b, 2019; Singh et al., 2020; Vannini et al., 2019; Yamashita et al., 2021). However, whether mitophagy is affected by mitochondrial disease and aging has remained undetermined.

Here, we report that abnormal mitophagy is a hallmark of mammalian mitochondrial muscle disease. We delivered adeno-associated viral (AAV) *mito*-QC-mitophagy reporter for mice with adult-onset mitochondrial myopathy (Deletor mice) (Tyynismaa et al., 2005). Deletor mice replicate the key molecular pathophysiology, stress, and treatment responses of human mitochondrial myopathy (Ahola et al., 2016; Forsström et al., 2019; Khan et al., 2017; Nikkanen et al., 2016; Pirinen et al., 2020; Suomalainen et al., 2011). Both Deletor mice and human patients with mitochondrial myopathy exhibit a slowly progressive RC deficiency with mosaic presence of muscle fibers in different stages of pathology (CNFs, RRFs, and fibers with normal morphology) and ultrastructural mitochondrial abnormalities. Deletor mice show progressive accumulation of heteroplasmic mtDNA rearrangements in their muscle, akin to mitochondrial myopathy patients and aging individuals (Cortopassi et al., 1992; Tyynismaa et al., 2005). Our study demonstrates the coexistence of fibers with induced or impaired mitophagy within the same diseased muscle. We show that the latter occurs in the most affected RRFs and involves mTORC1 activation, as mitophagy is partially rescued by rapamycin treatment. These results suggest that impaired mitophagy promotes the disease progression in aged muscle exhibiting mitochondrial dysfunction.

## Results

### Total levels of mitophagy are increased in muscle with mitochondrial dysfunction

**Figure 1:**
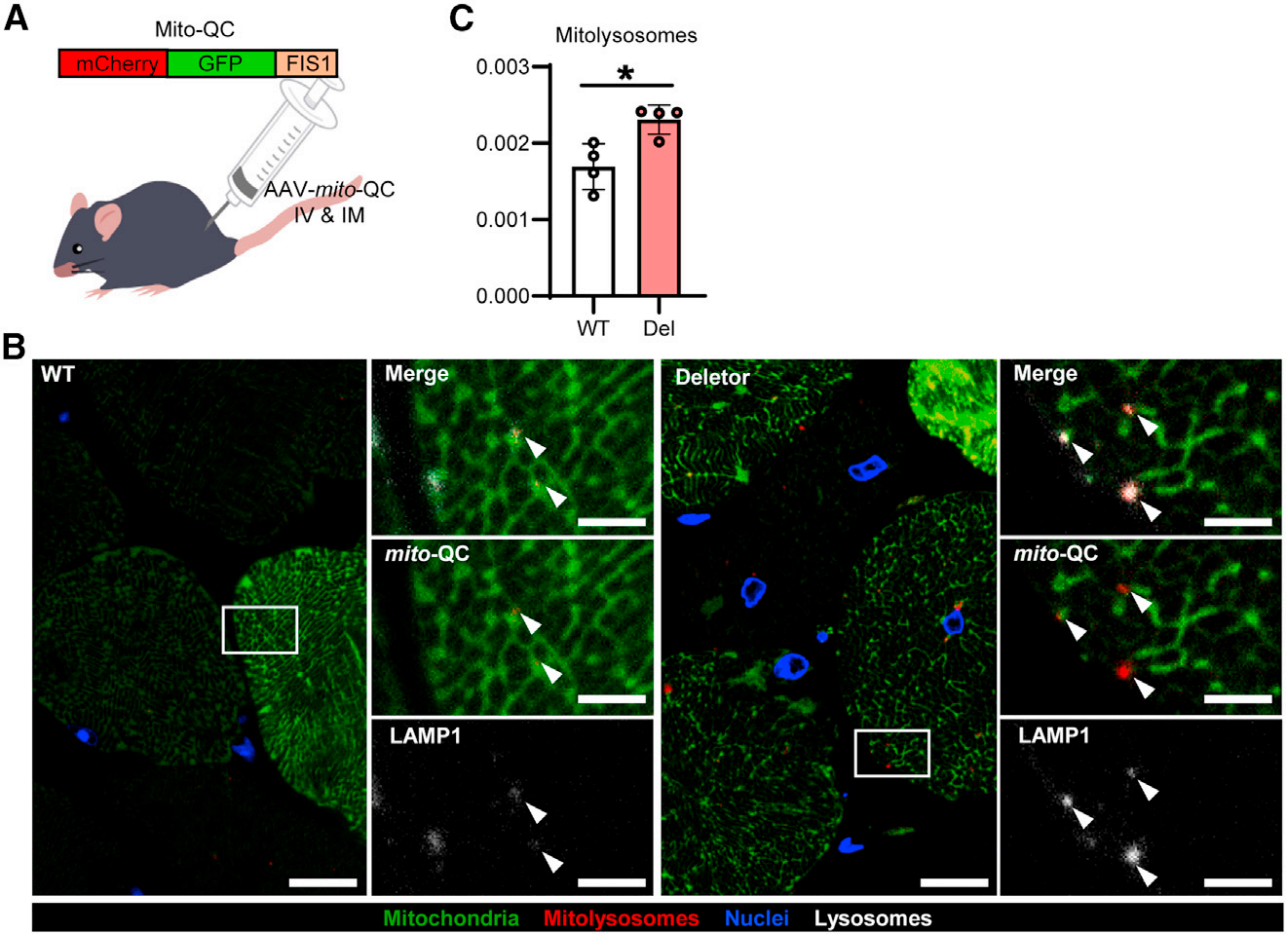
Aged Deletor muscle has elevated levels of mitophagy (A) Design of mitophagy reporter (*mito*-QC) in an adeno-associated virus vector construct (AAV*-mito*-QC; CAG promoter, serotype 9). IV, intravenous injection; IM, intramuscular injection. (B) Mitophagy in skeletal muscle (*QF*) of 24-month-old Deletor mice and wild-type (WT) littermates. Mitochondrial network and mitolysosomes visualized by *mito*-QC reporter. Representative confocal images of *mito*-QC signals with high-magnification enlargements (boxed with dashed lines). Mitolysosomes (arrowheads) were confirmed to be co-localized with lysosomal marker protein LAMP1 by immunostaining. Scale bars, 10 μm (lower magnification) and 3 μm (higher magnification). (C) Quantification of the number of mitophagy puncta in skeletal muscle, normalized by muscle fiber area. Deletor mice (Del) and their wild-type littermates (WT); ten regions of interest from each mouse, four different mice per genotype were quantified. Average values of each mouse are indicated as circles. Data are represented as mean ± SD. Student’s t test, ^∗^p < 0.05. See also Figures S1 and S2.

In order to assess *in vivo* mitophagy in mitochondrial myopathy, we established and applied the AAV vector carrying *mito*-QC (AAV*-mito*-QC) to the Deletor and wild-type (WT) littermate mice at 24 months of age (Figure 1A) and harvested skeletal (quadriceps femoris, *QF*) and cardiac muscle 3 weeks post-injection. Intravenous AAV injections efficiently transduced the heart but not skeletal muscle; thus, we combined these with intramuscular injections. This approach successfully illuminated both mitochondrial structure and mitophagy in Deletor and WT tissues (Figures 1B and S1A). Different fiber types showed similar transduction levels (Figure S1B), suggesting that our results are representative of the fiber-type distribution in *QF* muscle. This muscle was chosen for analyses because (1) in patients with muscle disease, vastus lateralis of the *QF* muscle is commonly used for diagnostic biopsy; (2) *QF* represents the proximal large muscles that are most affected in mitochondrial myopathy patients; and (3) *QF* accumulates COX-deficient fibers during aging in normal individuals after 50 years of age (Bua et al., 2006). The presence of *mito*-QC puncta in lysosomes was confirmed by immunohistochemistry to lysosomal associated membrane protein 1 (LAMP1) (Figures 1B and 1C). The total amount of mitolysosomes colocalizing with LAMP1 was significantly increased in the Deletor skeletal muscle. Deletor heart, with only occasional cardiomyocytes with mitochondrial RC deficiency, showed no difference in overall mitolysosome number (Figures S2A and S2B). However, in rare cardiomyocytes, clusters of mitolysosomes decorated nuclei in Deletors, but not in WTs (Figure S2A). Our results demonstrate widespread remodeling of mitophagy as a consequence of progressive dysfunction of the mitochondrial RC.

### Selective impairment of mitophagy in ragged-red fibers

**Figure 2:**
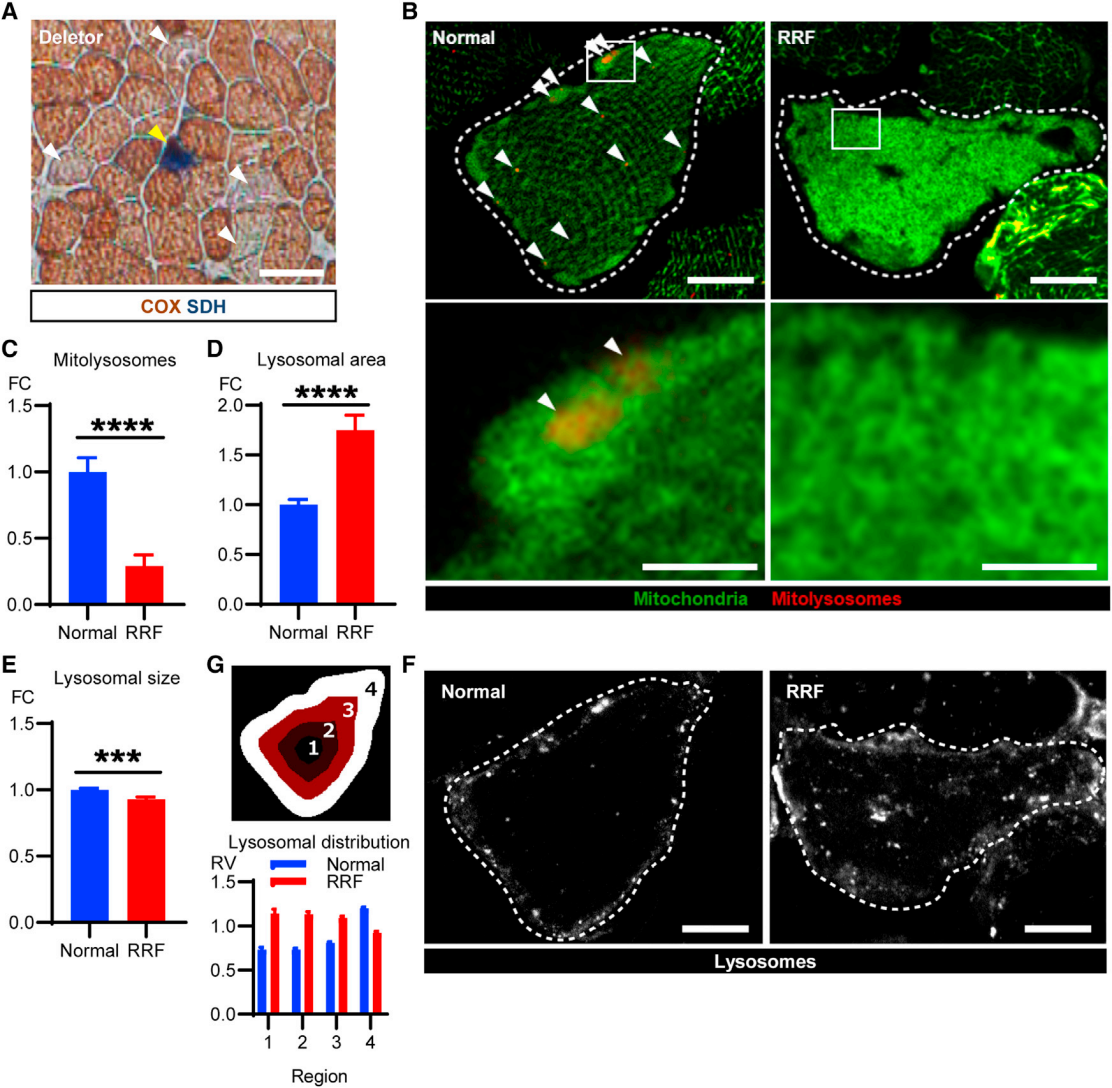
Defective mitophagy and aberrant lysosomal homeostasis in ragged-red fibers (RRFs) (A) Mitochondrial respiratory chain deficiency in Deletor mice. Histochemical activity analysis of cytochrome *c* oxidase (COX; Complex IV, partially mtDNA-encoded; brown precipitate) and succinate dehydrogenase (SDH; Complex II, nuclear encoded; blue precipitate) on frozen sections of *QF* muscle of Deletor mice. Yellow arrowhead, a COX-deficient, SDH-positive fiber with accumulated mitochondria (typical for a Deletor RRF, as reported in Khan et al., 2017). White arrowheads, fibers with partially decreased COX activity. Scale bar, 50 μm. (B) Mitochondrial network and mitophagy in a healthy muscle fiber and an RRF. Representative confocal images of *mito*-QC signals. Fiber borders are indicated as dashed lines. Arrowheads, mitolysosomes. Scale bars, 10 μm (lower magnification) and 1 μm (higher magnification). (C) Quantification of mitophagy. The total area of mitolysosomal puncta is normalized by muscle fiber area. (D and E) Quantification of lysosomes. The total area of LAMP1 positive puncta (lysosomes) is normalized by muscle fiber area (D) and average lysosomal size (E) in RRFs and morphologically normal fibers. (F) Lysosomal distribution in muscle fibers with normal morphology and RRFs. Representative confocal images of LAMP1 immunostaining in a RRF and a morphologically normal fiber. Fiber borders are indicated as dashed lines. Scale bars, 10 μm. (G) Quantification of the pattern of lysosomal distribution. Each muscle fiber was divided into four regions based on the distance from the center to the edge of the fiber (upper), and the number of lysosomes in each region were quantified and normalized by muscle fiber area of each region in RRFs and morphologically normal fibers. The results are presented as relative values (RV) to the average of the overall normal fibers (lower). Data are represented as mean ± SEM. All RRFs found in the *QF* muscles of four Deletors (89 fibers) and 100 morphologically normal fibers (25 fibers per mouse) were quantified. Student’s t test, ^∗∗∗^p < 0.001, ^∗∗∗∗^p < 0.0001.

The *QF* muscle of Deletor mice shows a mosaic pattern of mitochondrial dysfunction, with different stages of pathology, similar to patients with pathogenic mtDNA variants (Tyynismaa et al., 2005). This offered an excellent opportunity to assess mitophagy in the progression of RC deficiency in individual fibers. The RRFs account for 5%–10% of all muscle fibers in 2-year-old Deletors and were previously demonstrated to contain a high proportion of dysfunctional mitochondria (Tyynismaa et al., 2005). Consistent with this, confocal imaging of transduced RRFs revealed a remarkably disorganized mitochondrial network (Figures 2A and 2B). Despite the elevated overall levels of mitophagy in Deletor muscles (Figure 1C), comparative analysis revealed reduced mitophagy in RRFs compared with morphologically normal fibers (Figures 2B and 2C). These data suggest that selective impairment of mitochondrial turnover is a feature of mitochondrial disease progression.

Because mitophagy depends upon the efficient fusion of mitophagosomes with acidic endolysosomal structures, we next investigated whether impaired mitophagy in RRFs could reflect lysosomal dysfunction. RRFs had increased numbers of lysosomes (Figure 2D), yet the size of the individual lysosomes was decreased (Figure 2E). Furthermore, we observed their striking spatial disorganization: in muscle fibers with normal morphology, lysosomes were mainly localized in the subsarcolemmal region. However, in RRFs lysosomes were distributed across the fiber (Figures 2F and 2G), indicating aberrant lysosomal homeostasis in RRFs. Our collective findings suggest that RRF pathology is compounded by combined defects in lysosomal function and mitochondrial turnover.

### Mitophagy is increased in muscle fibers with central nuclei

**Figure 3:**
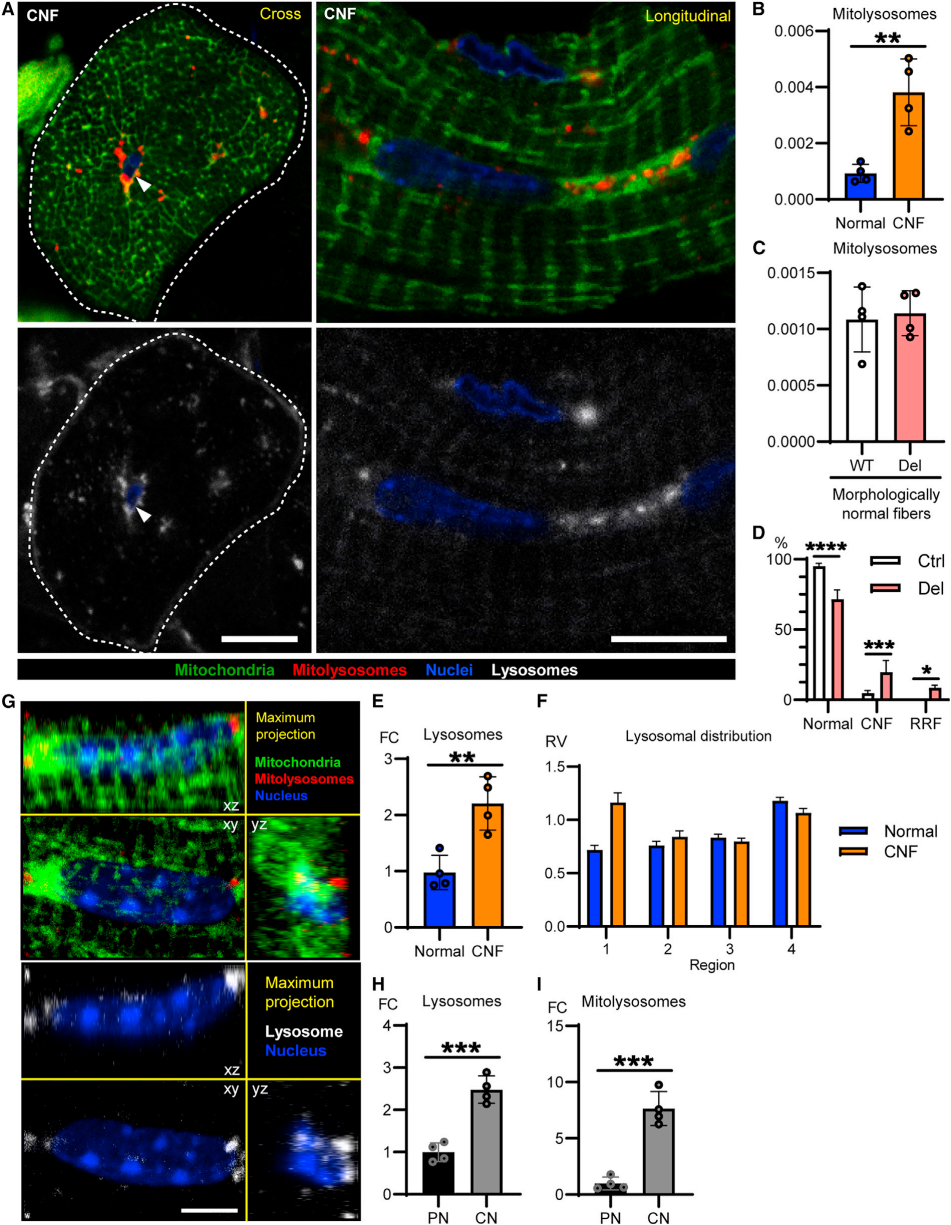
Centrally located nuclei (CNFs) are characterized by perinuclear mitophagy (A) CNFs in Deletor muscle, representative confocal images of *mito-*QC signals. Left: cross-sectional images. Right: longitudinal images. Fiber borders for CNFs are indicated as dashed lines. Arrowhead shows the centrally positioned nucleus surrounded by mitolysosomes. Scale bars, 10 μm. (B) Quantification of the number of mitolysosomal puncta normalized by muscle fiber area in CNFs and morphologically normal fibers in Deletor muscles. (C) Quantification of the mitophagy puncta normalized by muscle fiber area in morphologically normal fibers in Deletors and their wild-type (WT) controls. p = 0.7642. (D) Percentage of fibers classified as CNFs, ragged-red fibers (RRFs), and morphologically normal fibers in *QF* muscle of Deletor mice and WT littermates. (E) Quantification of the lysosomes (LAMP1-positive puncta) normalized by muscle fiber area in CNFs and morphologically normal fibers. (F) Quantification of the pattern of lysosomal distribution. Each muscle fiber was divided into four regions based on the distance from the center to the edge of the fiber, and the number of lysosomes in each region was quantified and normalized by muscle fiber area of each region in CNFs and morphologically normal fibers. The results are presented as relative values (RV) to the average of the overall normal fibers. Data are represented as mean ± SEM. (G) Representative z stack images of perinuclear region of central nuclei in Deletor muscle. Scale bar, 5 μm. (H and I) The number of lysosomes (H) and mitophagy puncta (I) in the perinuclear region of peripheral nuclei and central nuclei in Deletors. PN, peripheral nuclei; CN, central nuclei. Data are represented as mean ± SD unless otherwise stated. Twenty-five fibers of each class of fiber (B, C, E, and F), ten regions of interest (D), or 10 perinuclear regions of each type of nucleus (H and I) per mouse, four mice per group were analyzed. Average values of each mouse are shown as circles. Student’s t test, ^∗∗^p < 0.01, ^∗∗∗^p < 0.001, ^∗∗∗∗^p < 0.0001.

To investigate the discrepancy of why the total amount of mitochondrial turnover was increased in Deletor muscle even though mitophagy was decreased in RRFs, we quantified mitophagy in another class of pathological fiber, the centrally nucleated fibers (CNFs). CNFs have been used by pathologists to differentiate a muscle disease from a neurogenic disease (Dubowitz et al., 2007) and are also considered to have an active role in muscle repair and muscle disease pathogenesis (Folker and Baylies, 2013). Although CNFs are a sign of disease in Deletors, they still retain a structured mitochondrial network without mitochondrial accumulation, unlike RRFs (Figure 3A). In contrast to RRFs, we found an increased amount of mitolysosomes in CNFs compared with fibers with normal morphology (Figures 3A and 3B). In the morphologically normal fibers of Deletor muscle, mitophagy levels were comparable to WT controls (Figure 3C). The abundance of fibers with central nuclei is 2.3 times higher than that of RRFs in skeletal muscle of Deletor mice (Figure 3D), showing that the overall increase of mitophagy in Deletor muscle mainly reflects the increased mitophagy in the mildly affected CNFs. These data highlight that mitophagy is regulated by cell-autonomous signals and that induction or inhibition of mitophagy can occur even in adjacent fibers of the same muscle, depending on the severity of pathology in the individual fibers.

Lysosomal content was increased in CNFs (Figure 3E), suggesting the induction of lysosomal biogenesis in concert with mitophagy. The subsarcolemmal localization of lysosomes was not disturbed in CNFs, but abundance of lysosomes in the central area was increased (Figure 3F). Typically, an enrichment of lysosomes and mitophagy are evident in the perinuclear regions of peripheral nuclei of normal fibers. In CNFs, however, lysosomes and mitolysosomes were especially enriched in the region around the central nuclei (Figures 3G–3I).

### Phenotypic convergence of defective mitophagy in mice and patients

**Figure 4:**
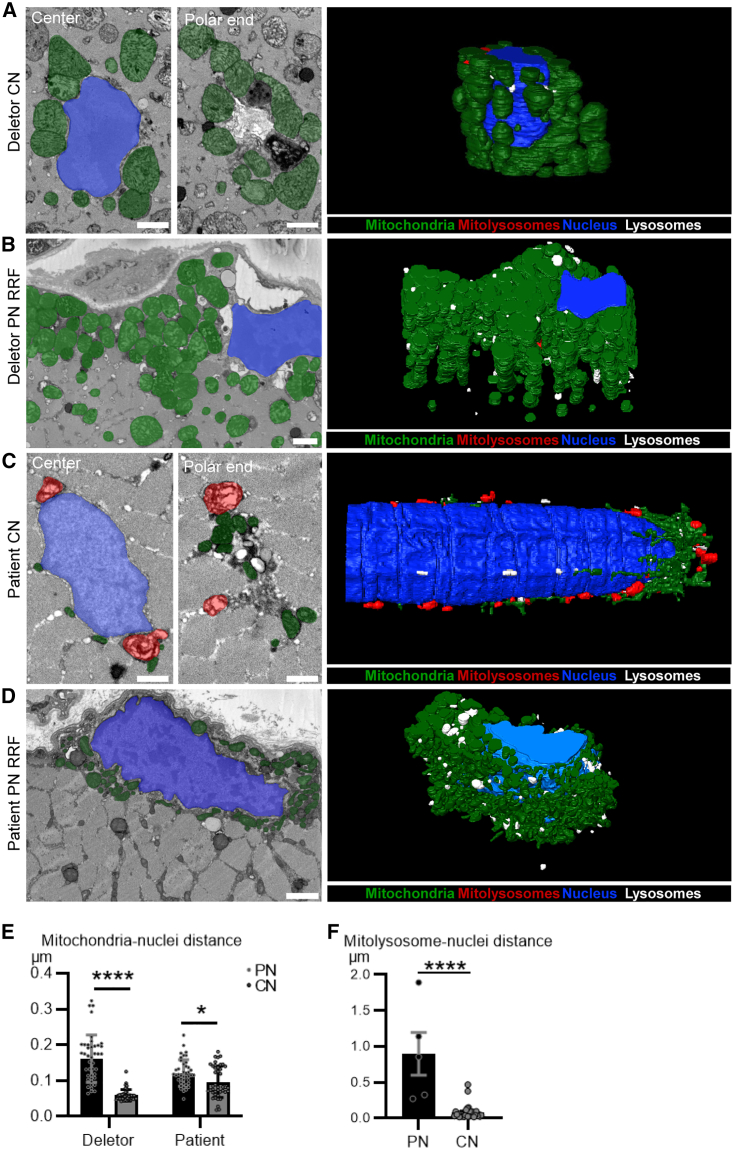
Polar positioning of mitophagosomes and close mito-nuclear contacts with central nuclei in Deletor mice and mitochondrial myopathy patients Serial block face scanning electron microscopy (SBFSEM) images of central nuclear and ragged-red fibers. (A) Deletor mouse muscle, central nucleus (CN). The polar end of the CN shows mitochondrial clusters and mitophagy. A three-dimensional reconstruction visualizes the spatial organization. (B) Deletor mouse muscle, peripheral nucleus (PN) in a ragged-red fiber (RRF). A three-dimensional reconstruction visualizes of the spatial organization. (C) Patient muscle (P1), mitochondrial myopathy: central nucleus. The polar end of the CN shows mitochondrial clustering and mitolysosomes. A three-dimensional reconstruction visualizes the 3D spatial organization. (D) Patient muscle (P1), mitochondrial myopathy: peripheral nucleus in an RRF. The polar end of the PN shows mitochondrial clustering. A three-dimensional reconstruction visualizes the 3D spatial organization. Scale bars, 500 nm. Pseudo-coloring: mitochondria (green), mitolysosomes (red), nuclei (blue), and lysosomes (white). (E) Quantification of the distances between mitochondria and peripheral/central nuclei in the three-dimensional electron micrographs of Deletor and patient (P1) muscles. Forty-two mitochondria per group were analyzed. (F) Quantification of the distances between mitolysosomes and peripheral nuclei (n = 5) or central nuclei (n = 29) in the three-dimensional electron micrographs of the patient muscle. Data are represented as mean ± SEM. Individual values are shown as circles. Student’s t test, ^∗^p < 0.05, ^∗∗∗∗^p < 0.0001. See also Figures S3 and S4 and Videos S1, **S2**, **S3**, and **S4**.

To further authenticate the spatial nature of the mitophagy phenotypes at the ultrastructural level, we performed high-resolution 3D imaging by serial block face scanning electron microscopy (SBFSEM) of muscle samples of both mice and human patients (Figures 4A–4D). In CNFs of the Deletor mice, the central nuclei were adorned with clusters of mitochondria, lysosomes, and mitolysosomes, linked by intimate contacts with the nuclear membrane (Figures 4A and 4E). Conversely, peripheral nuclei were devoid of such mito-nuclear contacts (Figures 4, S3, and S4; Figures S3A and S3B depict our ultrastructural criteria for judging the identity of the subcellular structures). In particular, mitochondria and mitophagic structures showed polar positioning in relation to the central nuclei (Figures 4A and S3; Video S1). Different from CNFs, RRF mitochondria accumulated in the subsarcolemmal region, but lysosomes were visible across the fiber, in agreement with our *mito*-QC data (Figure 4B; Video S2). In fibers with normal morphology, nuclear-mitochondrial contacts were low and lysosomes and degradative organelles occurred most frequently in the subsarcolemmal space (Figure S4A).

Video S1. 3D SBFSEM image of Deletor CNF, related to Figure 4

Video S2. 3D SBFSEM image of Deletor RRF, related to Figure 4

The Deletor mouse findings prompted us to perform SBFSEM on patient skeletal muscle samples. The findings (mitochondrial myopathy, caused by a single, large-scale mtDNA deletion) were remarkably similar to those in the Deletor muscle. The central nuclei showed mitochondrial clusters with mitophagy evident at opposite poles of a nucleus, and mitolysosomes and mitochondria formed close contacts with the nuclear membrane (Figures 4C, S3, and S4; Video S3 fibers with normal appearance showed peripheral nuclei closely located with lysosomes, degradation occurring specifically in the subsarcolemmal space, without close contact between nuclear and mitochondrial membranes (Figure S4B), mimicking our findings in mice. In addition, measuring the distance between mitochondria and nuclei in Deletors and patients demonstrated that mitochondria form a closer association with central nuclei than peripheral nuclei (Figure 4E), which was also true of mitolysosomes in patient muscle (Figure 4F). We then asked whether central nuclei also occurred in the RRFs of the patients. In the rare CNF-RRFs identified by SBFSEM, lysosomes and mitochondria surrounded the nucleus but mitophagosomes were absent (Figure S4C). Taken together, our findings demonstrate that aberrant mitophagy and lysosomal homeostasis characterize mitochondrial myopathy both in mice and human patients.

Video S3. 3D SBFSEM image of patient CNF, related to Figure 4

Video S4. 3D SBFSEM image of patient RRF, related to Figure 4

### Rapamycin partially restores mitophagy level in RRFs but has no effect on CNFs

**Figure 5:**
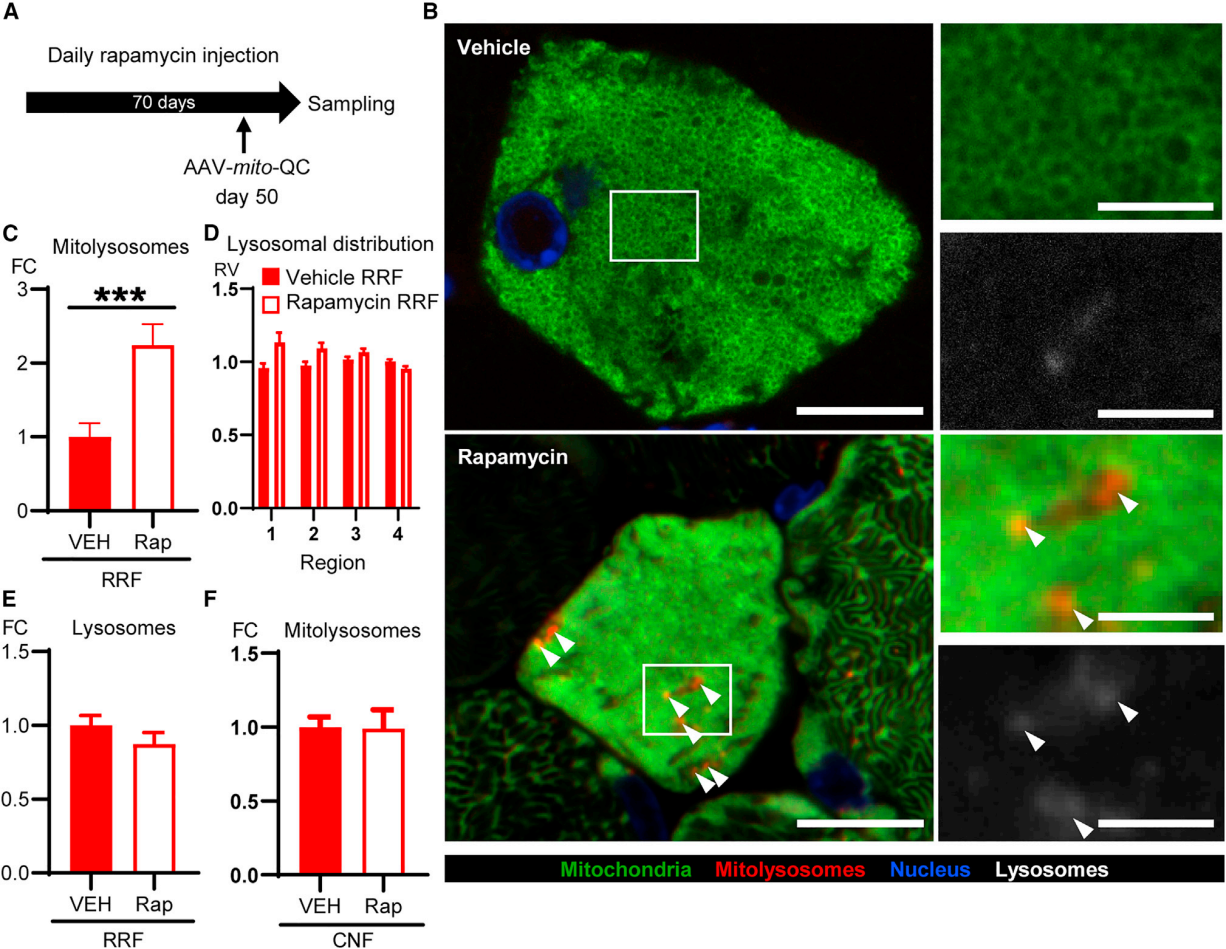
Rapamycin partially recovers mitophagy level in ragged-red fibers (RRFs) but has no effect on central nuclear fibers (CNFs) (A) Scheme of rapamycin treatment. Rapamycin or vehicle was daily injected for 70 days to four Deletor mice, respectively. AAV-*mito*-QC was injected on the 50th day of rapamycin injections. (B) Deletor muscle, RRFs; representative image of rapamycin’s effect on mitophagy. Scale bars, 10 μm (lower magnification) and 3 μm (higher magnification). Arrowheads, mitolysosomes. (C) Quantification of mitophagy in RRFs of Deletors after rapamycin injections (Rap) and vehicle injections (VEH). The total area of mitolysosomal puncta was normalized by muscle fiber area. (D) Quantification of the pattern of lysosomal distribution. Each muscle fiber was divided into four regions based on the distance from the center to the edge of the fiber, and the number of lysosomes in each region was quantified and normalized by muscle fiber area of each region in rapamycin or vehicle-treated RRF. The results are presented as relative values (RV) to the average of the overall vehicle-treated RRF. Data are represented as mean ± SEM. (E) Quantification of lysosomal abundance in Deletor RRFs with rapamycin injections. The total area of lysosomes was normalized by muscle fiber area. (F) Quantification of mitolysosomal abundance in Deletor CNFs with rapamycin injections and vehicle injections. The number of mitolysosomal puncta was normalized by muscle fiber area. Data are represented as mean ± SD unless otherwise stated. Forty fibers of each class of fiber, four mice per group were quantified. Student’s t test, ^∗∗∗^p < 0.001. See also Figure S5.

Previously, inhibition of mechanistic target of rapamycin (mTOR) complex 1 (mTORC1) by rapamycin was reported to partially rescue Deletor muscle pathology (Khan et al., 2017), decreasing the frequency of RRFs and activating macroautophagy, when assessed by total p62 and LC3B protein amounts (Khan et al., 2017). Here, we found that mTORC1 is activated in RRFs but not in CNFs (Figures S5A and S5B), suggesting a role of mTORC1 in RRF formation. To test whether this role was connected to mTORC1 inhibition of mitophagy, we treated 22-month-old Deletors with rapamycin (i.p. 8 mg/kg/day) for 70 days, and injected AAV*-mito*-QC on the 50th day of the rapamycin treatment (Figure 5A). Loss of mTORC1 activation in Deletor muscles was confirmed by loss of phosphorylation of S6 (pS6), ribosomal subunit and target of mTORC1 (Figure S5C). The mTORC1 inhibition was independent of the fiber type. We found that mTORC1 inhibition by rapamycin partially rescued mitophagy in RRFs (Figures 5B and 5C). However, rapamycin did not normalize the localization pattern of lysosomes (Figure 5D), and lysosomal number was not affected by rapamycin (Figure 5E). These results suggest that rapamycin did not recover mitophagy in RRFs through upregulating lysosomal synthesis in skeletal muscle with mitochondrial disease. In contrast to RRFs, rapamycin had no effect on the mitophagy level in CNFs (Figure 5F).

### Molecular factors behind the altered mitophagy in mitochondrial myopathy

**Figure 6:**
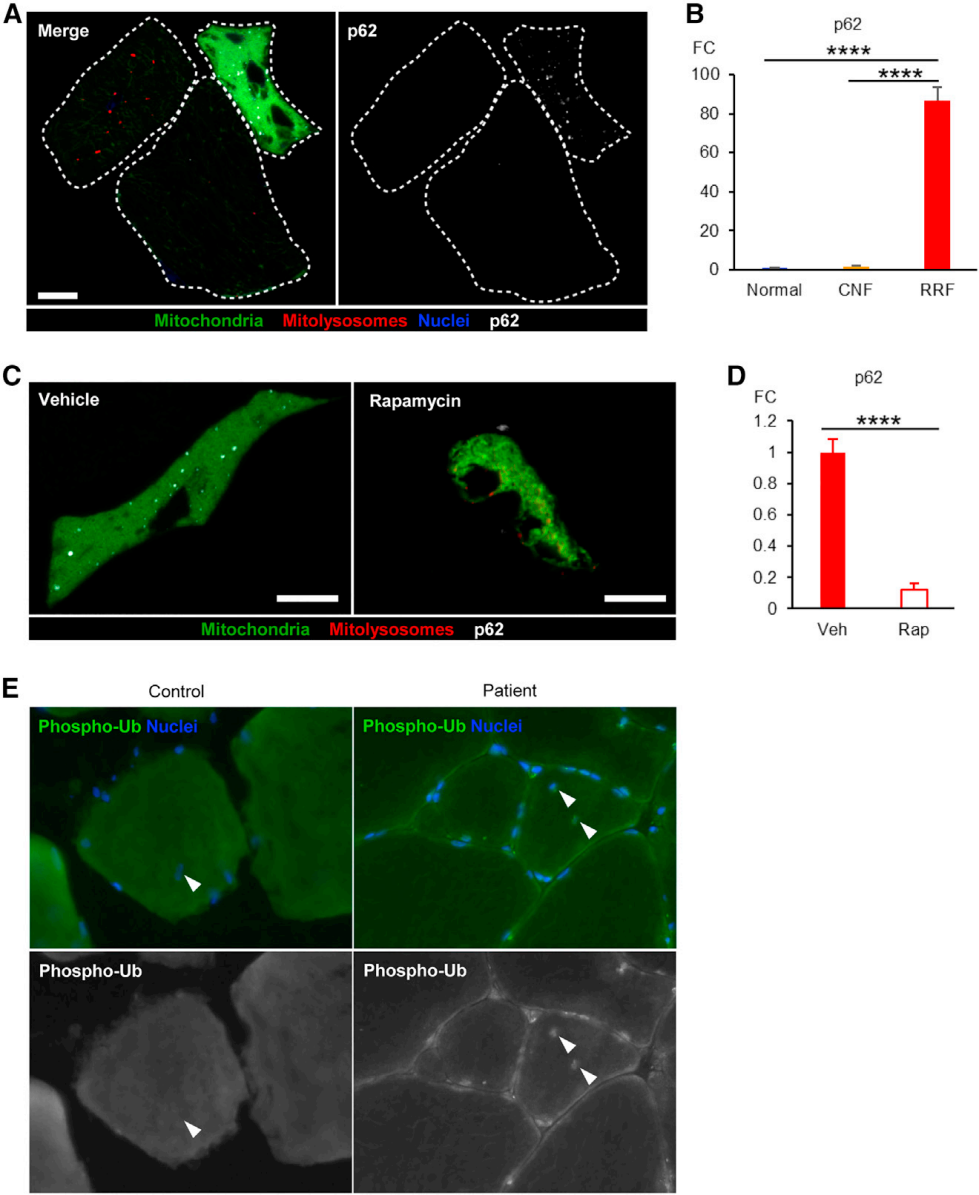
Molecular factors behind the altered mitophagy in mitochondrial myopathy (A) Representative images of p62 immunohistochemistry showing RRF-specific p62 accumulation in Deletor muscle with *mito*-QC signals. Fiber borders for CNF (left), normal fiber (middle), and RRF (right) are indicated as dashed lines. Scale bars, 10 μm. (B) Quantification of p62 abundance in normal fibers, CNFs, and RRFs in Deletor muscles, normalized by muscle fiber area. Thirty-six fibers per each class of fibers were quantified. The results are presented as relative values to the average of normal fibers. One-way ANOVA followed by Tukey’s multiple comparisons test, ^∗∗∗∗^p < 0.0001. No significant difference was found between normal fibers and CNFs (p = 0.9942). (C) Representative images of p62 immunohistochemistry showing rapamycin’s effect on RRF-specific p62 accumulation in Deletor muscle with *mito*-QC signals, Scale bars, 10 μm. (D) Quantification of p62 abundance in RRFs in Deletor muscles after rapamycin injections (Rap, n = 21) or vehicle injections (VEH, n = 36), normalized by muscle fiber area. The results are presented as relative values to the average of VEH. Student’s t test, ^∗∗∗∗^p < 0.0001. (E) Representative images of phosphorylated ubiquitin (phospho-Ub) immunohistochemistry showing perinuclear increase of phospho-Ub in patient muscles (P4), arrowheads indicate central nuclei. Scale bars, 50 μm. Data are represented as mean ± SD. See also Figure S6.

To elucidate the molecular factors behind the altered mitophagy in mitochondrial myopathy, we investigated known key factors for general macroautophagy and selective mitophagy. The autophagy activator, Beclin 1, was shown to be present more abundantly in RRFs in the muscle from patients with single, large-scale mtDNA rearrangements (Figure S6A). The findings of increased lysosomal amount and Beclin 1 together with decreased autophagic flux and stalled mitophagy indicate conflicting organellar homeostasis and turnover in RRFs. The autophagy adaptor protein p62 couples ubiquitin-tagged macroautophagy/mitophagy cargo with ATG8 proteins to promote autophagy (Katsuragi et al., 2015). Moreover, p62 was recently shown to be a mediator of Parkin-independent basal mitophagy in diseased liver (Yamada et al., 2018). p62 and LC3 were increased in RRFs in Deletor (Figures 6A and 6B) and patient muscles (Figure S6B), but not in normal fibers or CNFs, suggesting RRF-specific stalling of autophagic flux. The accumulation of p62 was partially rescued by rapamycin (Figures 6C and 6D), suggesting that both mitophagy and general macroautophagy were stalled in RRFs. Phosphorylation of ubiquitin, a modification dependent on PINK1/Parkin pathway (Kane et al., 2014; Kazlauskaite et al., 2014; Koyano et al., 2014), was increased compared with healthy individuals, especially in perinuclear regions, both peripheral and central nuclei (Figure 6E), suggesting that increased ubiquitin phosphorylation could participate in the increased mitolysosome content in CNFs.

## Discussion

The turnover of cellular components and organelles is essential to maintain tissue homeostasis. We show here that defective mitochondrial turnover underlies the progression of RC deficiency and pathology in skeletal muscle. Our data propose that mitophagic capacity contributes to manifestation of mosaic RC deficiency, a hallmark of normal aging muscle and mitochondrial disease. This mechanism is a plausible explanation also for the “threshold effect”—the mtDNA mutation load required to manifest a disease—a genetic characteristic of diseases associated with heteroplasmic mtDNA variants. Furthermore, our data enlighten the mechanisms underlying fibers with central nuclei and ragged-red fibers, two classic hallmarks of muscle histopathology. Interestingly, alterations in mitophagy constitute a key component of both pathological features, showing either restorative activation or stalling in a single muscle. Our data indicate that mitophagy activation is a promising treatment approach for aging-related mitochondrial dysfunction.

Mitochondrial quality control arises by several parallel mechanisms acting at several distinct levels, from targeted proteostasis to whole organelle turnover (Deshwal et al., 2020; Killackey et al., 2020; Narendra et al., 2008; Palikaras et al., 2018; Sugiura et al., 2014; Suomalainen and Battersby, 2018; Suomi and McWilliams, 2019). Targeted mitochondrial turnover, mitophagy, has been frequently suggested to contribute to disease, but direct evidence has been scarce. Indeed, the mere existence of heteroplasmic pathogenic mtDNA variants in skeletal muscle has been considered as evidence against existence of mitochondrial recycling in a postmitotic tissue. Here, we examined mitophagy in Deletor mice, a well-characterized mitochondrial myopathy model that accumulates heteroplasmic mtDNA mutations, manifests RC deficiency in a mosaic pattern in skeletal muscle and robustly recapitulates findings from human patients (Forsström et al., 2019; Nikkanen et al., 2016; Pirinen et al., 2020; Suomalainen et al., 2011; Tyynismaa et al., 2005, 2010). We show that mitochondrial myopathy can either promote or prevent mitophagy in a mosaic pattern, being increased in muscle fibers with subtle signs of disease (CNFs) but absent in the most severely affected fibers (RRFs). The markers for macroautophagy also pointed to mosaic decrease of autophagic flux in RRFs. Inhibition of mTORC1 activity, previously shown to be high in RRFs (Forsström et al., 2019; Khan et al., 2017), restored mitophagy (or macroautophagy) and autophagic flux in RRFs. Our results demonstrate an unexpected interplay between mTORC1 and mitochondrial turnover and dysfunction in mammalian mitochondrial muscle disease. Because mitophagy within adjacent muscle fibers is either elevated or deficient, our findings suggest that a cell-autonomous regulatory mechanism governs mitochondrial turnover in diseased muscle. These results further highlight the need for cell-specific *in vivo* studies in progressive pathology of mitochondrial dysfunction. Indeed, our quantitative analysis revealed that global levels of mitophagy in Deletor muscle were greater because of a high proportion of CNFs in the early disease stage, while the actual disease progression within RRFs was characterized by stalled mitophagy. In this case, bulk analysis of mitophagy would overlook the therapeutic potential of targeting mitophagy.

The precise contribution of CNFs to muscle disease pathology remains understudied. Central nuclei have been suggested to originate from satellite cell fusion, but why they are first centralized and then redistributed to subsarcolemmal space is unclear (Folker and Baylies, 2013). We show that CNFs have an intact mitochondrial network and lysosomal positioning, pointing to early-stage pathology. However, CNFs had greater numbers of mitolysosomes, characterized by peculiar focal positioning, especially close to the polar ends of the central nuclei. The mitochondria and mitolysosomes formed bridges between central nuclei, mimicking alternating beads on a string. Mitochondria also typically decorated expansive segments and invaginations of the nuclear membrane. The organellar clusters containing lysosomes, mitochondria, and mitolysosomes adjacent to the nucleus appeared similar to those previously reported in association with nucleophagy, in skeletal muscle with gene mutations affecting the nuclear membrane (Park et al., 2009). Our findings raise an intriguing possibility that mitochondria and lysosomes participate in remodeling/maturation of the central nucleus. This unusual crosstalk merits future investigation. Similar findings in Deletor mice and in patients with mitochondrial myopathy suggest mechanistic conservation.

Mosaic occurrence of RRFs is typically observed in muscle from patients with pathogenic mitochondrial tRNA variants or large-scale mtDNA rearrangements, i.e., mutational events affecting translation of mtDNA-encoded proteins. These gene defects are the most common causes of adult-onset mitochondrial diseases. MtDNA translation defects—but not those affecting structural components or assembly of RC complexes—also activate the mitochondrial integrated stress response (ISRmt), including remodeling of one-carbon cycle, induction of serine biosynthesis and metabokines FGF21 and GDF15 and activation of mTORC1 (Bao et al., 2016; Forsström et al., 2019; Kühl et al., 2017; Khan et al., 2017; Lehtonen et al., 2016; Nikkanen et al., 2016). Our findings *in vivo* unearth an important crosstalk between mitophagy, mTORC1 and ISRmt in disease. This disease-linked mTOR activity differs from basal PINK1-independent mitophagy, in which mTOR does not seem to contribute (McWilliams et al., 2018b). Our findings suggest that mitochondrial translation stress activates mitochondrial turnover, but when chronic, mTORC1 is activated, and both macroautophagy and mitophagy are inhibited, promoting disease progression.

We report that loss of lysosomal homeostasis coincides with defective mitophagy in RRFs. Lysosomes are widely distributed throughout the RRFs, instead of their typical localization in the subsarcolemmal region. The reciprocal absence of mitolysosomes and abundance of lysosomes in RRFs suggests defective recognition of damaged mitochondria with aberrant delivery to lysosomes or defective mitophagosome-lysosome fusion. Previously, mitochondrial dysfunction has been suggested to cause lysosomal deacidification upon RC deficiency, with enlargement of the lysosomes (Fernandez-Mosquera et al., 2019). We cannot exclude the possibility that RRF lysosomes may have hydrolytic deficiency which could contribute to impaired mitophagy. However, rapamycin treatment restored mitophagy, without seemingly rescuing lysosomal distribution in the myofiber, indicating existence of acidic, fusion-potent lysosomes in RRFs and/or partial restoration of lysosomal function by rapamycin. Mitophagy rescue by rapamycin was not complete, which may be a consequence of severely disrupted myocellular microtubular and contractile machinery in RRFs, affecting movement of the organelles for fusion, ATP deficiency hindering lysosomal delivery, or insufficient time of rapamycin intervention. Further studies of the mechanisms underlying the abnormal lysosomal distribution in RRFs may offer targets for intervention strategies for reversing or delaying the progression of mitochondrial dysfunction-related disorders of the muscle caused by mitochondrial dysfunction.

In contrast to its characterization as a mere autophagy adaptor protein, p62 promotes mitochondrial ubiquitylation and turnover also in a Parkin-independent fashion by recruiting Rbx1 and Keap1 (Yamada et al., 2018). They established a relationship between large mitochondrial size, p62 and stalled mitophagy in fatty mouse liver, where OPA1 depletion decreased mitochondrial size and rescued mitophagy. Similar to hepatocytes in fatty liver, RRFs are characterized by accumulation of large mitochondria, stalled mitophagy and accumulated p62, raising the possibility that the mitochondrial enlargement may also disrupt mitophagy. It is tempting to speculate that restoring mitochondrial volume could hold therapeutic potential for mitochondrial myopathy.

The mosaic manifestation of disease even in one tissue or organ points to the need of cell-specific *in vivo* analyses when assessing mitophagy. For this, novel optical mitophagy marker mouse lines, such as *mito*-QC mice (McWilliams et al., 2016), are ideally suited. However, crossing reporter mice to late-manifesting disease models is time consuming and has hindered examination of mitophagy in aging-related diseases. Our AAV9-mediated strategy overcomes this by enabling *mito*-QC transduction at any given age. AAV*-mito*-QC enables mitophagy studies also in other model animals, since AAV can infect various tissues in different animal species (Bevan et al., 2011; Davis et al., 2014; Federici et al., 2012; Zincarelli et al., 2008).

In conclusion, our evidence indicates stage-wise dynamics in mitophagic activity throughout mitochondrial disease progression in skeletal muscle, from early restorative to late-stage stalled states. *In vivo* visualization of mitophagy in Deletor mice alongside SBFSEM in patient samples demonstrated elevated mitophagy in CNFs and stalled in RRFs, even in adjacent cells of a muscle, in a cell-autonomous manner. Defective mitophagy in affected fibers may well explain why heteroplasmic pathogenic mtDNA variants are maintained and even accumulate in the muscle of patients’ and in normal aging. We propose that impaired mitophagy, lysosomal dysfunction, and mTORC1 activation conspire to induce RRF, a hallmark of mitochondrial myopathy. Further, we propose that CNFs are part of a repair/rescue response, with active role of mitophagy for remodeling of satellite-cell-derived central nuclei and/or remnant organelles. These data are mechanistically relevant beyond primary mitochondrial diseases, for different kinds of myopathies, muscle dystrophies, as well as inclusion body myositis, all characterized by secondary mitochondrial dysfunction and central nuclei. Finally, our data provide new therapeutic avenues to activate mitophagy in early-stage primary and secondary mitochondrial diseases of skeletal muscle, including aging-related muscle wasting.

### Limitations of study

The quantitative analysis of mitophagy and mitochondria by electron micrographs from muscle is semiquantitative because of the large size of muscle cells, ultrathin sections, and mosaic representation of disease.

The patients involved in the study harbored mtDNA deletions. Whether the findings apply to mitochondrial myopathies caused by other gene defects remains to be shown.

The mosaic cell-specific mitophagy phenotypes emphasize the complexity of mitochondrial pathophysiology. Therefore, achieving molecular mechanistic resolution using *in vivo* approaches is challenging. This issue is further compounded by the lack of reliable and specific tools for the detection of mitophagy pathway components in mouse tissue, a widely appreciated challenge in the field. Future studies using single-cell or spatial OMICs methods in these aged tissues will be required to further dissect the molecular mechanisms at play.

## STAR★Methods

### Key resources table

**Table:** 

REAGENT or RESOURCE	SOURCE	IDENTIFIER
**Antibodies**		

Rat-anti-LAMP1	Santa Cruz	Cat# sc-19992; RRID: AB_2134495
Rabbit anti-phospho-S6	Cell Signaling Technology	Cat# 4858; RRID: AB_916156
Mouse anti-p62	Abcam	Cat# ab56416; RRID: AB_945626
Rabbit anti-Beclin 1	StressMarq	Cat# SPC-600D
Mouse anti-MTCO1	Abcam	Cat# ab14705; RRID: AB_2084810
Mouse anti-VDAC1	Abcam	Cat# ab14734; RRID: AB_443084
Rabbit anti-phospho-Ubiquitin (Ser65)	Millipore	Cat# ABS1513-I; RRID: AB_ 2858191
Rabbit anti-LC3	Cell Signaling Technology	Cat# 4108; RRID: AB_2137703
Goat anti-rat IgG Alexa fluor 633	Thermo Fisher Scientific	Cat# A-21094; RRID: AB_141553
Anti-mouse IgG2a CF405S	Sigma	Cat# SAB4600476
Anti-rabbit IgG Alexa fluor 488	Thermo Fisher Scientific	Cat# A-11008; RRID: AB_ 143165
Anti-mouse IgG2b fluor Alexa 546	Thermo Fisher Scientific	Cat# A-21143; RRID: AB_ 2535779
Anti-mouse IgG2a Alexa fluor 647	Thermo Fisher Scientific	Cat# A-21241; RRID: AB_ 141698
VECTASTAIN Elite ABC-Peroxidase Kit	Vector Laboratories	Cat# PK-6101; RRID: AB_2336820
Mouse/Rabbit specific HRP/DAB detection IHC kit	Abcam	Cat# ab64264

**Chemicals, peptides, and recombinant proteins**		

Paraformaldehyde	Sigma Aldrich	Cat# P6418
HEPES	Sigma Aldrich	Cat# H4034
Sucrose	Thermo Fisher Scientific	Cat# 10634932
Phosphate buffered saline	Sigma Aldrich	Cat# P5493
Isopentane	Alfa Aesar	Cat# 19387
O.C.T. compound	Sakura	Cat# Z4583
Formalin	FF-Chemicals	Cat# 1209010
BSA	Thermo Fisher Scientific	Cat# BP9703
Triton X-100	Sigma Aldrich	Cat# X100
Vectashield Antifade Mounting Medium	Vector Laboratories	Cat# H-1000
Prolong Gold Antifade Mountant	Thermo Fisher Scientific	Cat# P10144
Hoechst 33342	Thermo Fisher Scientific	Cat# 62249
DRAQ5	Abcam	Cat# ab108410
Citric Acid	Sigma Aldrich	Cat# 251275
Hydrogen Peroxide	Sigma Aldrich	Cat# 516813
Avidin/biotin blocking kit	Vector Laboratories	Cat# SP-2001
Horse serum	Thermo Fisher Scientific	Cat# 26-050-088
TBS	Thermo Fisher Scientific	Cat# 11300499
3,3’-diaminobenzidine tetrahydrochloride (DAB)	Sigma Aldrich	Cat# D9015
Rapamycin	LC Laboratories	Cat# R-5000
DMSO	Sigma Aldrich	Cat# D8418
PEG-400	Sigma Aldrich	Cat# 202398
Catalase	Sigma Aldrich	Cat# C40
Cytochrome c	Sigma Aldrich	Cat# C2506
Disodium Phosphate	Sigma Aldrich	Cat# S5136
Monosodium Phosphate	Sigma Aldrich	Cat# S9638
Acetone	Thermo Fisher Scientific	Cat# A060617
Aluminum pin	TAAB	Cat# G312
Aspartic acid	Sigma Aldrich	Cat# A9256
Cacodylate buffer	Agar Scientific	Cat# R1104
Copper grids	Gilder Grids	Cat# GA1500-C3
Epoxy resin	TAAB	Cat# T030
Glutaraldehyde	TAAB	Cat# G003
Gold coating	Agar Scientific	Cat# B7370
Lead Nitrate	Sigma Aldrich	Cat# 228621
Potassium ferrocyanide	Sigma Aldrich	Cat# 244023
Osmium tetroxide	Agar Scientific	Cat# AGR1024
Thiocarbohydrazide	Sigma-Aldrich	Cat# 223220
Toluidine blue	TAAB	Cat# SD211
Silver glue	Agar Scientific	Cat# G3648
Uranyl acetate	Agar Scientific	Cat# AGR1260A

**Experimental models: Organisms/strains**		

Tg/ACTB/twnk-p.353-365-dup/BL6	Suomalainen Lab	N/A

**Software and algorithms**		

Microscopy Image Browser	Belevich et al. (2016)	http://mib.helsinki.fi/
Amira	Thermo Fisher Scientific	https://www.thermofisher.com/amira-avizo
Ilastik 1.3.3	Berg et al. (2019)	https://www.ilastik.org/
CellProfiler 4.06	McQuin et al. (2018)	https://cellprofiler.org/
Prism 8.4.3	GraphPad Software	https://www.graphpad.com/scientific-software/prism/

**Other**		

LSM 880 Confocal Laser Scanning Microscope with Airyscan	Zeiss	N/A
Axioplan 2 Universal Microscope	Zeiss	N/A
Axio Imager M1 microscope	Zeiss	N/A
Sigma SEM	Zeiss	N/A
3View system	Gatan	N/A
Pelco BioWave Pro+ Microwave Processing System	Ted Pella	N/A
HT 7800 TEM	Hitachi	N/A
AMT 40 CCD Camera	Deben	N/A

### Resource availability

#### Lead contact

Further information and requests for resources and reagents should be directed to the Lead Contact, Anu Suomalainen (anu.wartiovaara@helsinki.fi).

#### Materials availability

This study did not generate novel reagents.

### Experimental model and subject details

#### Ethical approvals

Animal experiments were performed according to guidelines approved by the ethical board of State province Office for Animal Experimentation of Finland, following the principles of European Union Directive. The collection and use of human materials were performed with prior informed consent, following the ethical approval granted by Newcastle and North Tyneside Research Ethics Committees (REC: 18/NI/0199 and 16NE/0267). Control human muscle was acquired with prior informed consent during routine anterior cruciate ligament surgery following ethical approval granted by Newcastle and North Tyneside Research Ethics Committees (REC: 12/NE/0394).

#### Mouse Model

Mitochondrial myopathy model Deletor mouse carrying a transgene with a dominant mutation (duplication of amino acids p.353-365) in *Twnk* was generated in C57BL/6J background and has been characterized in detail in previous studies (Tyynismaa et al., 2005, 2010). Wild type littermate mice were used as controls. Mice were housed in cages in controlled room at 22°C and 12 h light/dark cycle with *ad libitum* access to food and water and their health status was checked daily by the responsible animal caretakers at the Laboratory Animal Centre of the University of Helsinki. All the mice in this study were given rodent diet with 11% fat, 65% carbohydrate, 24% protein of total calory content (Altromin Spezialfutter GmbH & Co. KG). The male Deletor mice and their controls that were used for the AAV-*mito*-QC transduction with or without daily rapamycin injections were 24 or 22 months old.

#### Subjects

A small piece of fresh skeletal muscle sample from a 44-year-old female patient (P1), with mitochondrial myopathy resulting from a single, large-scale heteroplasmic mtDNA deletion was collected in saline soaked gauze and processed immediately for SBFSEM. For staining experiments muscle samples from single, large-scale mtDNA deletion patients (n=4, P2-5) were frozen in liquid nitrogen cooled isopentane and stored at -80°C (Table 1). Diagnostic muscle biopsies were collected via needle biopsy under local anesthesia from the vastus lateralis of *quadriceps femoris* muscle.

**Table 1:** Clinical details of patient cohort

Patient	Sex (M/F)	Age at biopsy (years)	Clinical presentation	mtDNA deletion breakpoints	Heteroplasmy (%)
P1	F	44	N/A	m.9754-15567	27
P2	F	22	myopathy, diabetes, atypical retinopathy	m.8577-12986	72
P3	F	72	CPEO	m.12112-14412	60
P4	F	53	CPEO, myopathy	m.9486-13723	87
P5	F	36	CPEO	m.7117-14631	33

Muscle biopsies from these cases were used in the EM, immunofluorescent, and immunohistochemical labeling experiments. Deletion breakpoints are numbered according to the revised Cambridge reference sequence (NC_012920.1). CPEO, chronic progressive external ophthalmoplegia; N/A, not available; M, male; F, female.

### Method details

#### Adeno-associated virus carrying *mito*-QC

DNA construct for *mito*-QC was established in a previous study (Allen et al., 2013). The *mito*-QC construct was inserted into the pSubCAG-WRPE vector plasmid. For AAV*-mito*-QC serotyp-9 production, HEK293 cells were transfected with the plasmid. The virus suspension was collected, filtered, concentrated, and stored at –80°C until use.

#### Virus administration and sample preparation

24-month-old male Deletor mice and their wild type littermates were injected with AAV*-mito*-QC intramuscularly (9 x 10^11^ v.p.) and intravenously (4 x 10^12^ v.p.). The cardiac and *QF* muscles were collected three weeks after the AAV*-mito*-QC administration. For immunofluorescence and confocal microscopy, tissues were fixed in 3.7% paraformaldehyde at pH 7.0 in 0.2 M HEPES for 24 hours, cryoprotected in 30% (w/v) sucrose in phosphate buffered saline at 4°C and frozen in liquid nitrogen-cooled isopentane after embedded in O.C.T. compound (SAKURA). For phospho-S6 immunohistochemistry, the skeletal muscle samples were fixed in 10% buffered formalin and embedded in paraffin.

#### Mouse muscle immunostaining

For mouse immunofluorescence experiments, 5 μm thick frozen tissue sections were incubated with rat anti-LAMP1 antibody (sc-19992, Santa Cruz) or mouse anti-p62 antibody (ab56416, Abcam) for 15 hours after 60 minutes blocking with 5% BSA in 0.4% Triton X-100/PBS. Sections were then incubated with anti-rat IgG Alexa fluor 633 (A-21094, Thermo Fisher Scientific) or anti-mouse IgG2a CF405S (SAB4600476, Sigma) for 60 minutes. Slides were mounted with Vectashield Antifade Mounting Medium (H-1000, Vector) after nuclear counter staining with Hoechst 33342 (62249, Thermo Fisher Scientific) or DRAQ5 (ab108410, Abcam). Confocal images were acquired using a laser-scanning confocal microscope (LSM880, Zeiss). For phospho-S6 immunohistochemistry, 12 μm thick paraffin embedded sections were incubated with rabbit anti-phospho-S6 antibody (4858, Cell Signaling Technology) for 15 hours after citric acid antigen retrieval in a microwave for 2 minutes, H_2_O_2_ treatment, avidin-biotin blocking and 30 minutes blocking with 2% horse serum/TBS. Detection was done with VECTASTAIN Elite ABC-Peroxidase Kit (PK-6101, Vector) according to the manufacturer’s instructions followed by chromogen DAB staining, nuclear counter staining with hematoxylin and imaging by light microscopy (Axioplan 2 Universal Microscope, Zeiss)

#### Human muscle immunostaining

Cryosections of 10 μm thickness were collected two per slide. For immunofluorescent detection of Beclin 1 the staining protocol was adapted from Rocha et al. (2015). Briefly sections were air dried and fixed in ice cold 4% PFA for 3 minutes, before permeabilization in an ascending and descending methanol gradient (70% 10 minutes, 95% 10 minutes, 100% 20 minutes, 95% 10 minutes and 70% 10 minutes). Sections were washed in TBST before blocking with 10% NGS for 1 hour at room temperature. Rabbit anti-Beclin1 (SPC-600D, StressMarq), mouse anti-MTCO1 (ab14705, Abcam) and mouse anti-VDAC1 (ab14734, Abcam) were diluted in 10% NGS and incubated at 4°C overnight. Sections were washed in TBST, before incubation for 2 hours at 4°C with secondary antibodies; anti-rabbit IgG Alexa fluor 488 (A-11008, Thermo Fisher Scientific), anti-mouse IgG2b fluor Alexa 546 (A-21143, Thermo Fisher Scientific) and anti-mouse IgG2a Alexa fluor 647 (A-21241, Thermo Fisher Scientific) all at 1:200 and DAPI at 1:400). Sections were washed in TBST and mounted in Prolong Gold Antifade Mountant (P10144, Thermo Fisher Scientific). For anti-phospho-Ub sections were fixed for 3 minutes in methanol free 4% PFA, washed in PBS, before blocking in 1xPBS/5%NGS/0.3%TritionX-100) for 1 hour. Rabbit anti-phospho-Ub (Ser65) antibody (ABS1513-I, Millipore) was diluted 1:50 in 1xPBS / 1% BSA/ 0.3% TritonX-100 and incubated overnight at 4°C. Sections were washed in PBS before incubation with anti-rabbit IgG Alexa fluor 488 (1:200) and DAPI (1:400) in 1xPBS /1% BSA /0.3% TritonX-100 for 2 hours at 4°C. Sections were then washed in PBS before mounting in Prolong Gold Antifade Mountant. For LC3 immunohistochemistry a Abcam Mouse/Rabbit specific HRP/DAB detection IHC kit (ab64264, Abcam) was used following the manufacturer's protocol, with an ascending and descending methanol gradient and 1 hour protein block with 10% NGS after the Hydrogen Peroxidase Block and before the kit Protein Block. Rabbit anti-LC3 antibody (4108, Cell Signaling Technology) was used at 1:50 in 10% NGS and incubated overnight. Images were taken by Axio Imager M1 microscope with both a monochrome AxioCam MRm and color AxioCam MRc (Zeiss).

### Sequential COX/SDH histochemistry

Frozen muscle sections (12 μm thick) were reacted to demonstrate both cytochrome c oxidase (COX) and succinate dehydrogenase (SDH) activities using standard protocols (Old and Johnson, 1989). In brief, sections were incubated in phosphate buffer with 3, 3 –diaminobenzidine, catalase, cytochrome c and sucrose at 37°C and then in phosphate buffer with nitro-blue tetrazolium and sodium succinate at 37°C. Sections were dehydrated, air dried and imaged by light microscopy (Axioplan 2 Universal Microscope, Zeiss or Axio Imager M1, Zeiss).

#### Rapamycin administration

Rapamycin (#R-5000, LC Laboratories) was dissolved in DMSO to 100 mg/mL and diluted in 5% PEG-400 to final concentration of 1.2 mg/mL, sterile filtered and stored at –80°C. 22 months old Deletor mice and their wild type littermates were intraperitoneally injected with 8 mg/kg/day rapamycin or the same volume of vehicle for 70 days. On the 50th day of the rapamycin injections, each mouse was intramuscularly (9 x 10^11^ v.p.) and intravenously (4 x 10^12^ v.p.) injected with AAV-*mito-*QC. The tissues were collected three weeks after the virus admirations.

#### Serial Block Face Scanning Electron Microscopy sample preparation and imaging

Skeletal muscle samples were prepared for SBFSEM as previously described (Faitg et al., 2020). Briefly, samples were lightly teased apart in 0.1M Sorenson’s phosphate buffer to allow penetration of solutions between the muscle fibers. The sample was then cut into 1 mm^3^ pieces before fixation in 2% glutaraldehyde with 0.1 M Sorenson’s buffer (pH 7.3) at 4°C for 10 minutes. Samples were washed with three changes of Sorenson’s buffer in a microwave (3 x 150 W for 40 seconds per wash). Samples were then incubated either with cytochrome c oxidase (COX) activity reaction mixture (5 mM 3’,3 diaminobenzidine tetrahydrochloride (DAB) 1000 μM cytochrome c and 0.2 μg catalase in 0.1 M phosphate buffer, pH 7.0) for 2 hours at 37°C or Sorenson’s buffer for 2 hours at 4°C. As previously demonstrated, COX histochemistry does not perturb mitochondrial morphology or sample structure (Faitg et al, 2020). Following the incubation, the sample was washed three times as above and then processed using a heavy metal protocol adapted from Wilke et al. (2013). Samples were immersed in 3% potassium ferricyanide with 2% osmium tetroxide for 1 hour at room temperature. Tissue was then placed in filtered 0.1% thiocarbohydrozide (TCH) for 20 minutes and then 2% osmium tetroxide for 30 minutes. Samples were left in 1% urinyl acetate overnight at 4°C. The samples were washed for three times 5 minutes in ddH_2_O and immersed in lead aspartate solution (0.12g lead nitrate in 20 ml aspartic acid) for 30 minutes at 60°C. Samples were then dehydrated in a graded acetone series (25%, 50%, 75%, 3 x 100%) in the microwave (300 W, 3 minutes per step) and then immersed in increasing concentrations (25% to 100%) of TAAB 812 hard resin in acetone. The samples were embedded in 100% fresh resin and left to polymerize at 60°C for 36-48 hours. Blocks were then trimmed and sectioned for TEM to identify areas of interest for SBF-SEM. For each region of interest, a series of images (400 images per stack) were captured, at 70 nm sectioning thickness and with dimensions of 2000 x 2000 pixels (pixel size 0.07 μM x 0.07 μM). Nuclei, mitochondria, lysosomes and mitophagy like structures were segmented in Microscopy Image Browser (Belevich et al., 2016). Models were exported as Amira Mesh files, opened in Amira for 3D visualization and videos and screen shots exported.

### Quantification and statistical analysis

For the quantification of confocal images, each pixel of the images was classified into mitochondria (GFP positive), red-only puncta (GFP negative, mCherry positive and LAMP1 positive), lysosome (LAMP1 positive), p62 accumulation (p62 positive) or nuclei (Hoechst positive) and all the rest were considered as “other compartments”. Then all the pixels were quantified by Ilastik 1.3.3 software (Berg et al., 2019) and CellProfiler 4.06 software (McQuin et al., 2018). For the EM quantification, distances between the mitochondria and nuclei were measured in Microscopy Image Browser, using the measure length tool. Due to differences in total volume for each region of interest a total of 46 mitochondria per region were analyzed to allow unbiased comparison. The minimum distance was also measured between peripheral and mitolysosmes (n=5) and central nuclei and mitolysosomes (n=29) in the patient. For all measurements the minimum distance between the two objects was exported. All image analyses were performed in a blinded manner. Data are presented as means ± SD unless otherwise noted. Comparisons between two groups were performed by an unpaired two-tailed Student’s t test. Multi-comparisons were performed by one-way ANOVA following Tukey’s multiple comparisons test. The p-value < 0.05 was considered to be statistically significant. No methods were used to determine whether the data met assumptions of the statistical approach. Statistical parameters were reported either in individual figures or corresponding figure legends. All statistical analysis was performed using Prism 8.4.3 Software (GraphPad Software).

## Data Availability

• All data reported in this paper will be shared by the lead contact upon reasonable request. • No new code has been generated in this study. • Any additional information required to reanalyze the data reported in this paper is available from the lead contact upon request.

## Supplemental information

Document S1. Figures S1–S6

Document S2. Article plus supplemental information
