# Supplementary material for: Mosaic dysfunction of mitophagy in mitochondrial muscle disease
Source: Cell Metab. 2022 Feb 1;34(2):197–208.e5. doi: 10.1016/j.cmet.2021.12.017 (PMC8815775; doi:10.1016/j.cmet.2021.12.017)
Supplement: Document S1. Figures S1–S6 [file mmc1.pdf]

Cell Metabolism, Volume *34*

Supplemental information

Mosaic dysfunction of mitophagy

in mitochondrial muscle disease

Takayuki Mito, Amy E. Vincent, Julie Faitg, Robert W. Taylor, Nahid A. Khan, Thomas G. McWilliams, and Anu Suomalainen

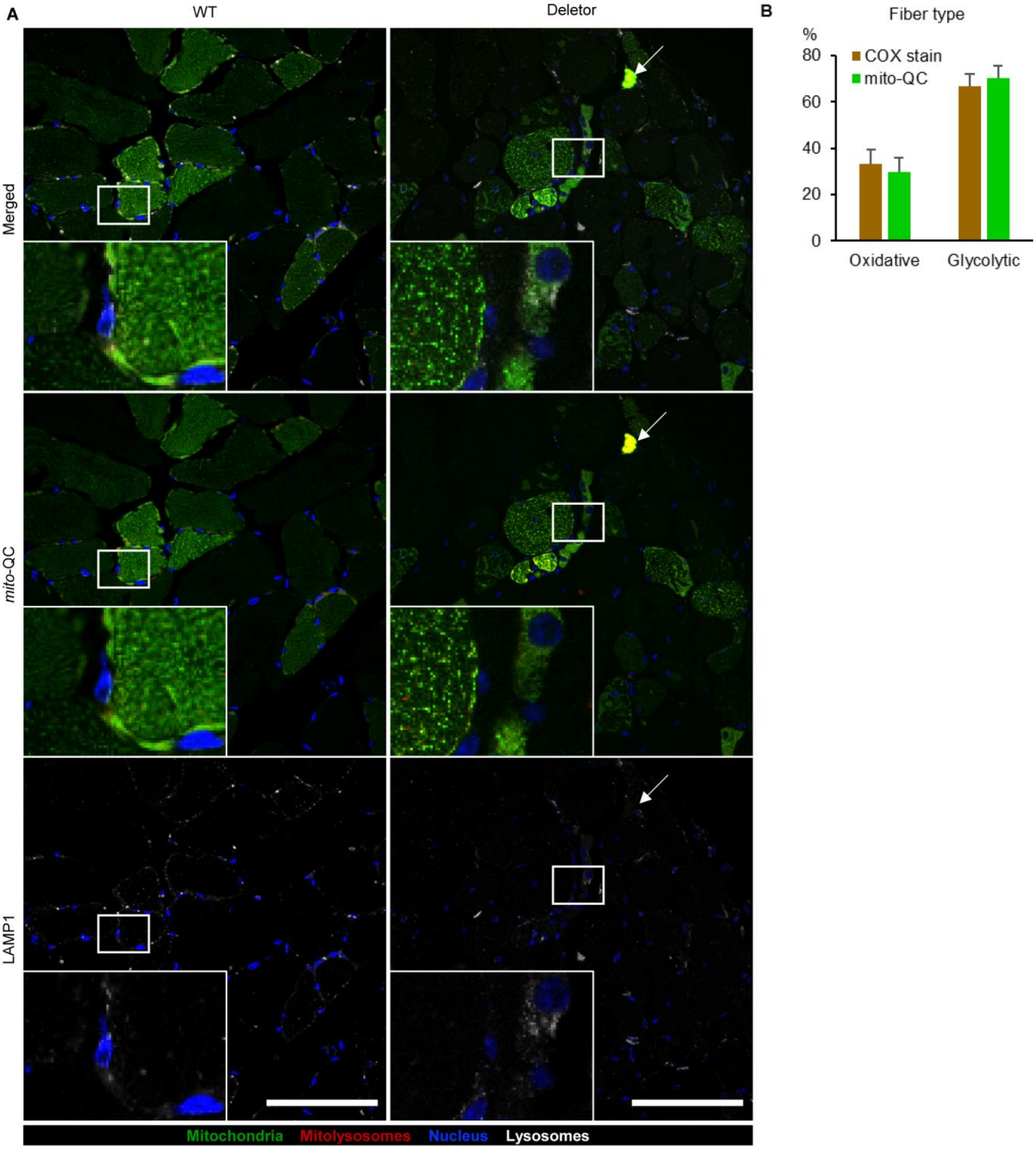
A
WT
Deletor
Merged
mito-QC
LAMP1
Mitochondria
Mitolysosomes
Nucleus
Lysosomes
B
Fiber type
%
80
60
40
20
0
COX stain
mito-QC
Oxidative
Glycolytic

Figure S1. An overview of in vivo transduction efficiency of AAV-mito-QC in the skeletal (*QF*) muscle, Related to Figure 1.

(A) Representative confocal images of *mito*-QC signals in Deletor muscles and their wild type (WT) controls. Although variable transduction levels are a common feature of viral-mediated expression *in vivo*, the ratiometric properties of *mito*-QC enable an assessment of mitolysosomes irrespective of expression levels. For quantification we employed a conservative approach, including only the positive fibers with discernible mitochondrial networks, omitting those with saturated signal (arrow). Scale bars, 100 µm.

(B) Analysis of transduction levels between different fiber types. Fiber type composition of *mito*-QC transduced fibers was quantified based on their morphology (presence/absence of the sub-sarcolemmal mitochondria; high in slow oxidative and low in glycolytic fast fibers) and compared with that of total *QF* muscle visualized by COX staining. No significant difference in *mito*-QC transduction efficiency was found in fibers representing different metabolic types. Data are represented as mean ± SD. Student’s t-test.

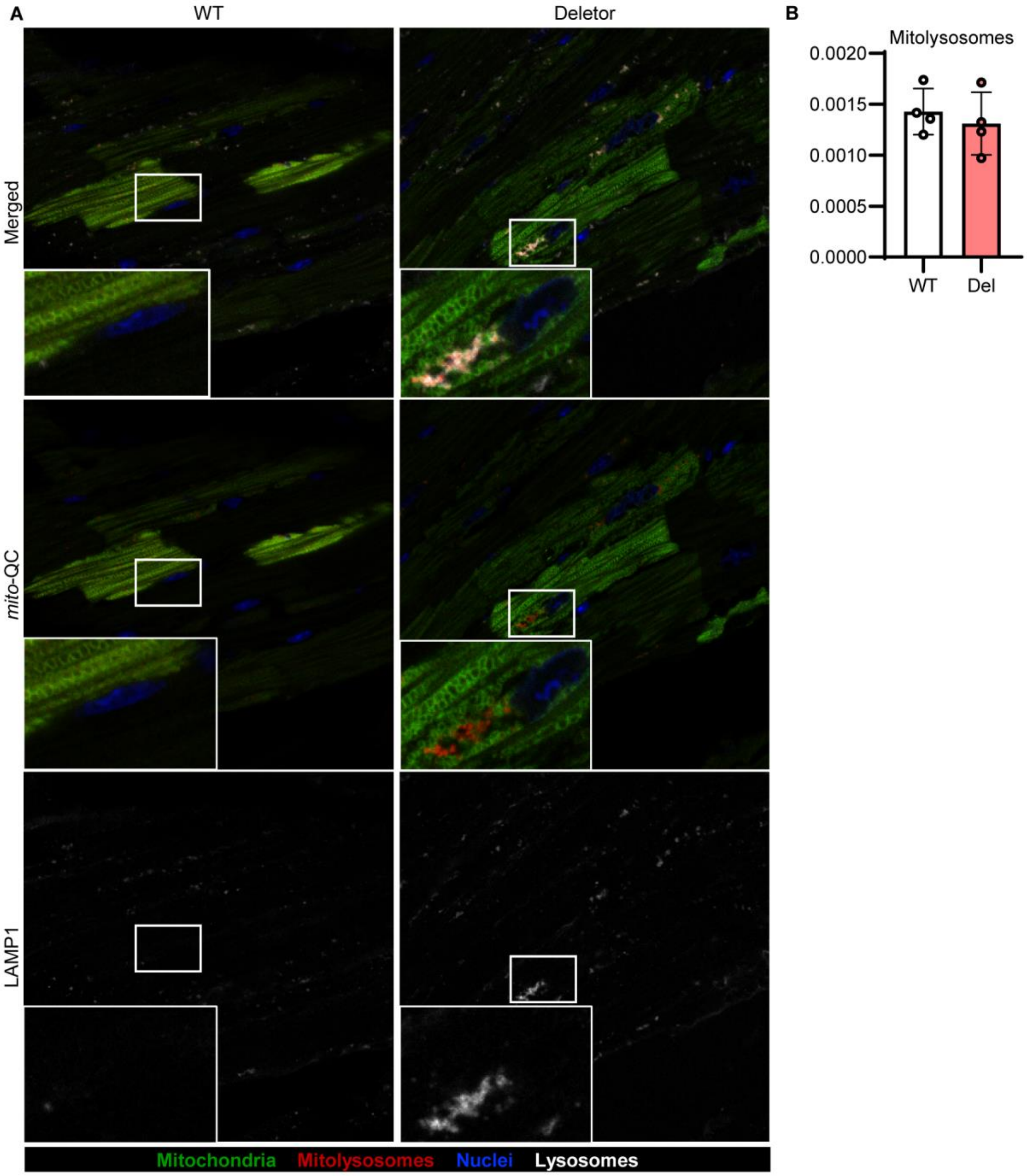

Figure S2. Cardiac mitophagy, Related to Figure 1.

(A) Cardiac muscle, mitophagy in 24-month-old Deletor and wild type (WT) mouse cardiac muscle. Representative confocal images of *mito*-QC signals. Scale bar, 50 μm.

(B) Quantification of mitolysosomes in the heart, normalized by muscle fiber area. 10 regions of interest per mouse, four different mice per group were quantified. Average values of each mouse are shown as circles. Data are represented as mean ± SD. Student's t-test.

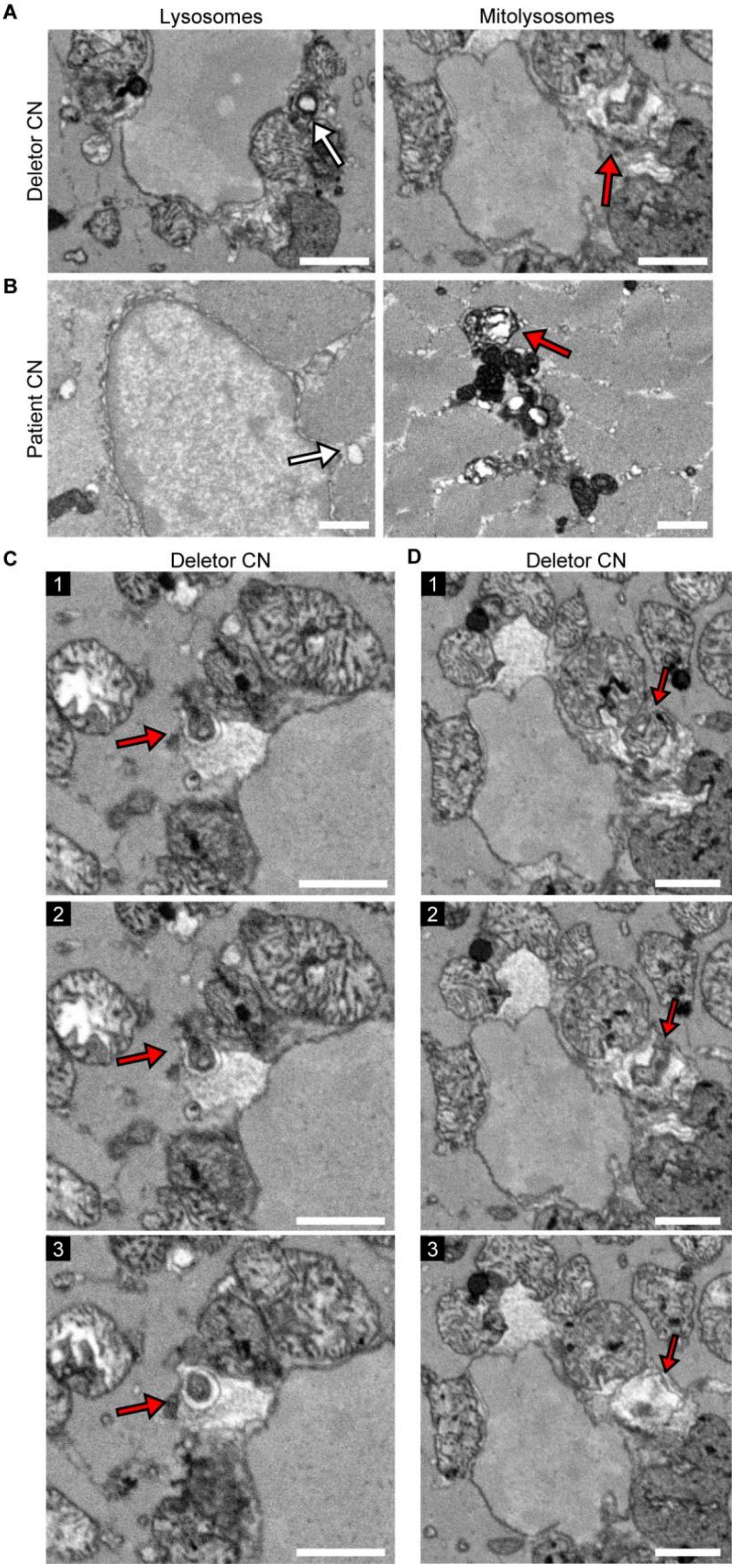
A
Lysosomes
Mitolysosomes
Deletor CN
B
Patient CN
C
Deletor CN
D
Deletor CN
1
2
3
1
2
3

Figure S3. Representative ultrastructural images of lysosomes and mitolysosomes; the basis of classification of these subcellular structures, Related to Figure 4.

(A) Deletor mouse, skeletal muscle. 2D SBFSEM images of a central nucleus (CN). Lysosome indicated by a white arrow (left) and a mitolysosome indicated by a red arrow (right). Scale bar, 1 µm.

(B) Patient, mitochondrial myopathy, skeletal muscle. 2D SBFSEM images of a central nucleus. Lysosome (white arrow, left); mitolysosome (red arrow, right). Scale bar 0.5 µm.

(C and D) Serial z-stack images showing mitophagosomes (red arrow) adjacent to Deletor CNs. Mitochondria and mitolysosomes form close contacts with CNs. Scale bar 1 µm.

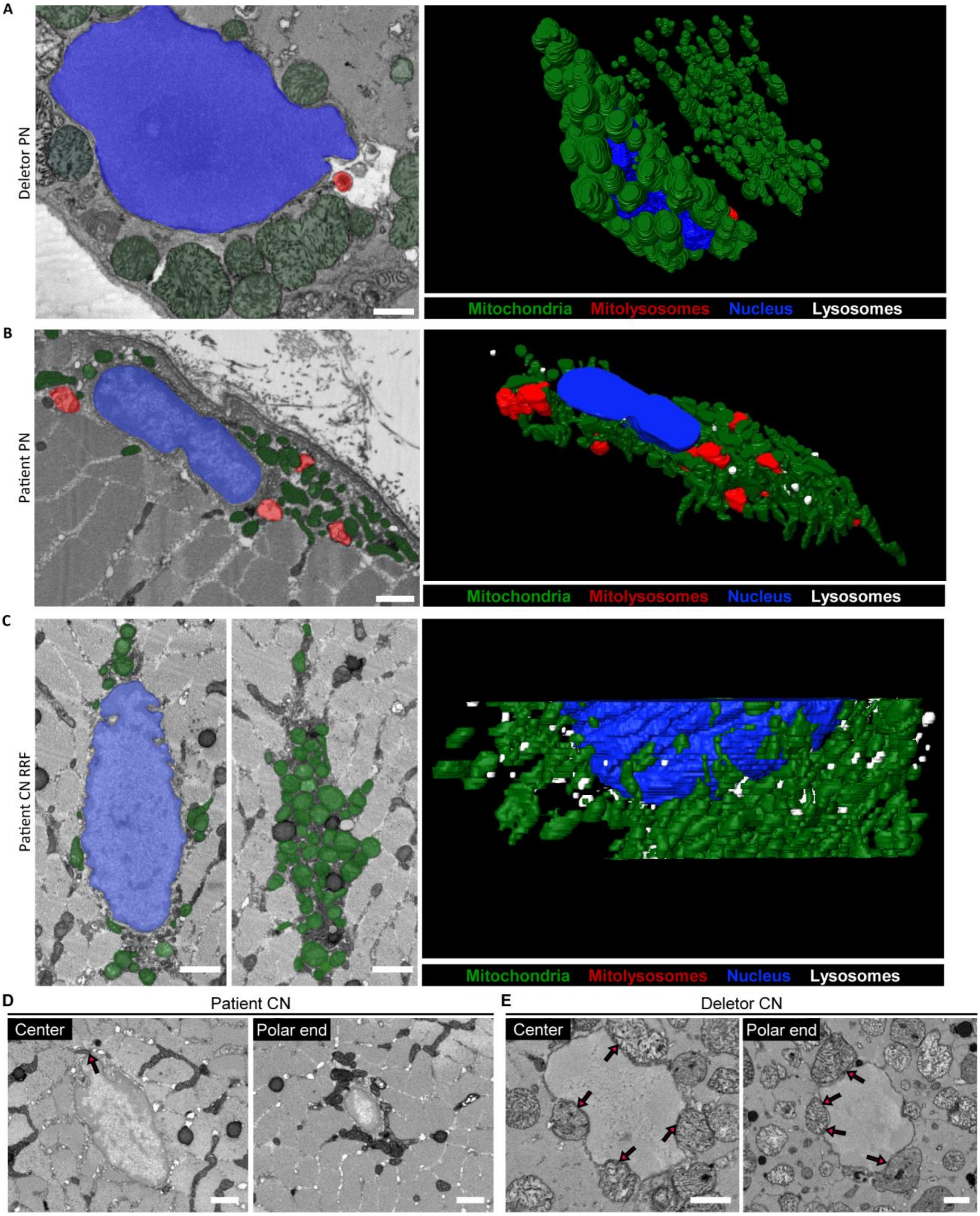

A
Deletor PN
Mitochondria
Mitolysosomes
Nucleus
Lysosomes
B
Patient PN
Mitochondria
Mitolysosomes
Nucleus
Lysosomes
C
Patient CN RRF
Mitochondria
Mitolysosomes
Nucleus
Lysosomes
D
Patient CN
Center
Polar end
E
Deletor CN
Center
Polar end

Figure S4. Mito-nuclear contacts are typical for central nuclei in Deletor mouse and patients. Related to Figure 4.

Serial block face scanning electron microscopy images.

(A) Deletor mouse muscle, morphologically normal fiber peripheral nucleus (PN). A pseudocoloured EM image (left) and a three-dimensional reconstruction (right).

(B) Patient, mitochondrial myopathy, skeletal muscle. A PN from a normal muscle fiber. Pseudocoloured EM image (left) and a three-dimensional reconstruction (right).

(C) Patient, mitochondrial myopathy, skeletal muscle. A central nucleus (CN) in ragged red fiber (RRF). The polar end of the nucleus shows mitochondrial clustering. Right: a three-dimensional reconstruction. Scale bar, 500 nm.

(D and E) Serial z-stack images of CNs showing mito-nuclear contact sites around CNs in skeletal muscles of a mitochondrial myopathy patient (D) and a Deletor mouse (E). Pink arrows show mito-nuclear contact sites. Mitochondria are highly abundant around the CN of Deletor mice with a high surface area of contact sites in comparison to the patient. Scale bar, 1 µm.

Pseudo-coloring: Mitochondria (green), Mitolysosomes (red), Nucei (blue) and Lysosomes (white).

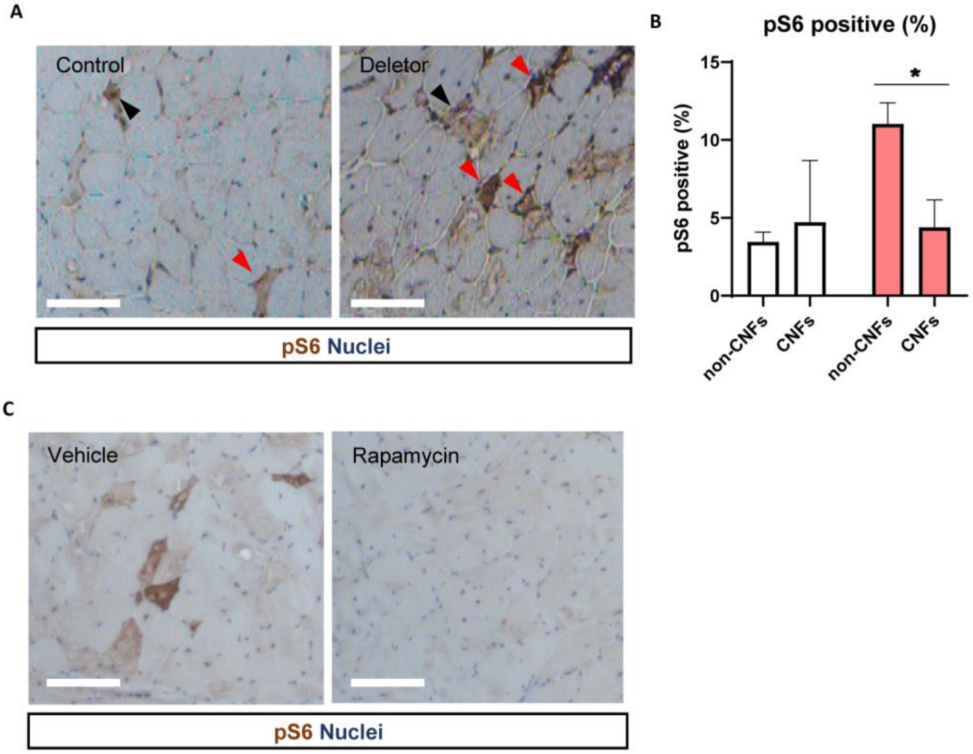

Figure S5. mTOR activation in Deletor muscle is inhibited by rapamycin, Related to Figure 5.

(A). Representative images of phospho-S6 (pS6, mTORC1 target) immunohistochemistry, Deletor muscle. Black arrowheads and red arrowheads indicate pS6 positive CNFs and non-CNFs, respectively. Scale bars, 50 μm.

(B) Quantification results of (A); CNFs and non-CNFs were classified based on the presence / absence of the internal nuclei (hematoxylin staining) and pS6 positive / negative fibers were counted to calculate the percentage of pS6 positive fibers in CNFs and non-CNFs, respectively. Deletor (pink bars) and wild type (white) mouse muscle. Two regions of interest per mouse, four mice per group. Data are represented as mean ± SD. Student's t-test, * $p < 0.05$.

(C) pS6 immunohistochemistry showing the loss of mTOR activation after rapamycin injections; Deletor muscle. Scale bars, 50 μm.

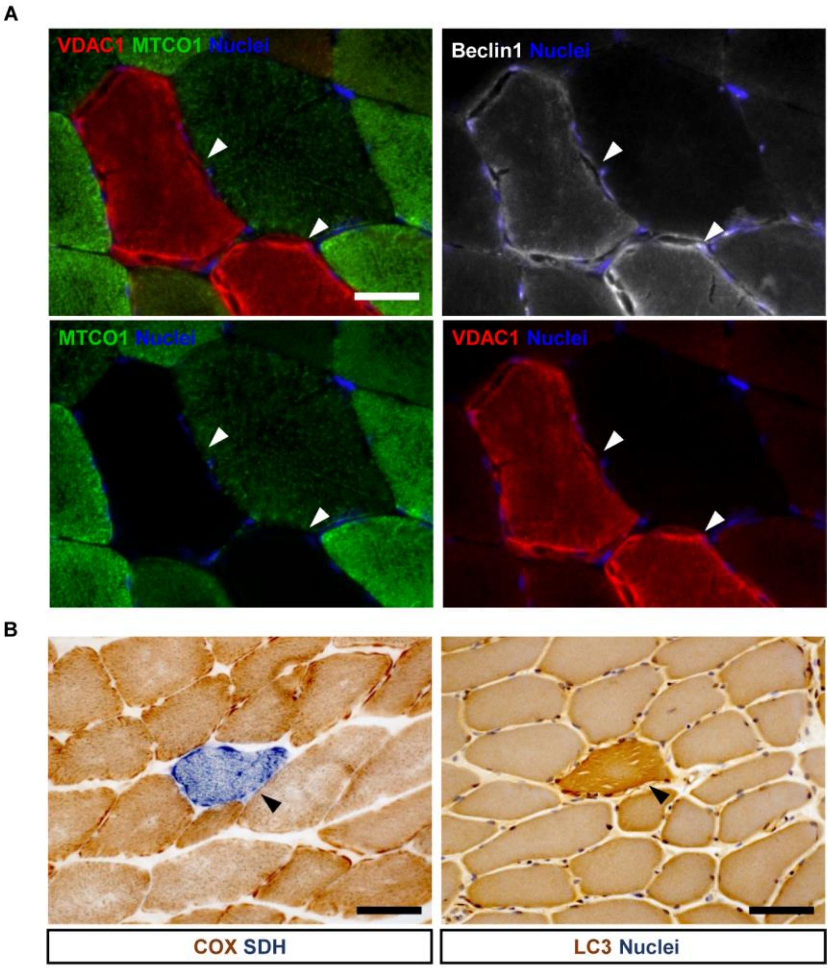

Figure S6. Molecular factors behind the altered mitophagy in mitochondrial myopathy, Related to Figure 6.

(A) Representative images of Beclin1, VDAC1 (mitochondrial marker, nuclear genome coded) and MTCO1 (mitochondrial marker, mtDNA coded) immunohistochemistry showing RRF specific increase of Beclin1 in patient muscles (P4). MTCO1 negative fibers with elevated VDAC1 signal indicate RRFs (arrowheads) with accumulated dysfunctional mitochondria. Scale bars, 50 μm.

(B) Representative images of COX/SDH histochemistry and LC3 immunohistochemistry on serial sections showing RRF (arrowhead) specific increase of LC3 in patient muscles (P4). Scale bars, 100 μm.
